# Simple models of genomic variation in human SNP density

**DOI:** 10.1186/1471-2164-8-146

**Published:** 2007-06-06

**Authors:** Raazesh Sainudiin, Andrew G Clark, Richard T Durrett

**Affiliations:** 1 Department of Statistics, University of Oxford, Oxford, OX1 3TG, UK; 2 Department of Mathematics, Cornell University, Ithaca, New York 14853, USA; 3 Department of Molecular Biology and Genetics, Cornell University, Ithaca, New York 14853, USA

## Abstract

**Background:**

Descriptive hierarchical Poisson models and population-genetic coalescent mixture models are used to describe the observed variation in single-nucleotide polymorphism (SNP) density from samples of size two across the human genome.

**Results:**

Using empirical estimates of recombination rate across the human genome and the observed SNP density distribution, we produce a maximum likelihood estimate of the genomic heterogeneity in the scaled mutation rate *θ*. Such models produce significantly better fits to the observed SNP density distribution than those that ignore the empirically observed recombinational heterogeneities.

**Conclusion:**

Accounting for mutational and recombinational heterogeneities can allow for empirically sound null distributions in genome scans for "outliers", when the alternative hypotheses include fundamentally historical and unobserved phenomena.

## Background

Understanding the population-genetic forces behind the observed variation among human genome sequences is vital to deciphering the genetic causes of phenotypic variation among humans. The phenomena that influence the density of human SNPs include (1) variation-introducing events that are empirically observable, such as, point-mutations, recombinations, and activities of various transposable elements that may result from the counteraction of various DNA damage and repair pathways [[1], for e.g.], as well as (2) genealogy-affecting events that are historical and generally unobserved, such as population dynamics, population structure, and natural selection. A biological understanding of the observed genomic variation in SNP density, by means of explicit population-genetic models of coalescence in the presence of recombination and mutation, must incorporate any interplay among the heterogeneities in the above phenomena. Here we strive for an empirically sound understanding of the observed human SNP density, as determined by a genome-wide alignment of two different consensus sequences, by accounting for the empirically observable mutational and recombinational heterogeneities under the simplest model of population history (selectively-neutral, constant-sized, random-mating). The two sequences are the NCBI human genome sequence and the sequence produced by Celera Genomics [2]. Our SNP density data were obtained from first aligning the Celera consensus sequence to the NCBI assembly and then counting the number of SNPs in bins of 100 kb (100,000 base pairs), as was done in section 6 of the above study [2]. Next, we build simple models for the distribution of SNP density from random samples of size 2 from a locus that is 100 kb in length. Our objective is to explain as much of this simple measure of diversity as possible, under empirically sound null hypotheses that include coarse-grained, genome-wide measurements of recombinational variation.

## Results

Two approaches toward modeling are taken. The first approach is descriptive and employs hierarchical Poisson models to obtain better fits than the homogeneous Poisson distribution used earlier [2]. Insights gained from the first approach inform the second approach. The second approach is non-descriptive and population-genetic with biologically interpretable parameters. It employs mixtures of SNP densities simulated under the coalescent with different mutation and recombination rates to obtain a better fit to the observed SNP density distribution. This approach introduces heterogeneity into the coalescent-based simulation of SNP density that was shown to produce a poor fit under the assumptions of genome-wide homogeneity and equality of mutation and recombination rates [2]. The simple closed-form expressions used in the paper are elementary results in coalescent theory [3,4].

### Descriptive Hierarchical Poisson Models

Let Λ and *T *be the parameters in the mass function of a Poisson distribution given by Pr(*X *= *x*|Λ*T*) = *e*^-Λ*T*^(Λ*T*)^*x*^/*x*!. The random variables Λ and *T *are generally *proxies *for relative mutation rate and the sum of branch lengths of the coalescent trees for all the non-recombining segment(s) of the 100 kb locus, respectively. In other words, *T *is a proxy for the sum of the branch lengths of the ancestral recombination graph (ARG-size) of our sample of size 2 at a locus that is 100 kb long. The random variable *X *represents the count of SNPs in contiguous 100 kb intervals from an alignment of two human genomes. In this hierarchical scheme, heterogeneities are modeled by the Gamma and Beta probability density functions (*PDF*s), *G*(*γ*_1_, *γ*_2_) and *B *(*β*_1_, *β*_2_), respectively, as described in Methods. We chose the Gamma distribution *G*(*γ*_1_, *γ*_2_) to model *T *for the following reasons. When there is no recombination, the depth of the coalescent tree of two samples is exponentially distributed with rate parameter 1, i.e., *G*(1,1). And when there are *n *sites with free recombination in between them, the sum of the *n *independent and exponentially distributed depths is *G*(*n*, 1). Thus, *T *is only a mathematically convenient proxy for the ARG-size of our sample of size 2, since *T *~ *G*(*γ*_1_, *γ*_2_) does not explicitly capture the distribution of ARG-size for intermediate levels of intra-locus recombination among sites at our locus. We use the relatively flexible Beta family on [0, 1] to model, Λ which is a proxy for relative mutation rate. The Poisson distribution for SNP density follows from the assumption of the infinitely-many-sites mutational model under selective neutrality, where mutations hit a site at most once according to the product of the total length of the site-specific coalescent tree and the site-specific relative mutation rate. Therefore, such hierarchical Poisson models are merely descriptive, as they are built via mathematically convenient Beta and Gamma distributed random variables Λ and *T *that act as proxies for the relative mutation rate and the ARG-size, respectively.

**Figure 1:**
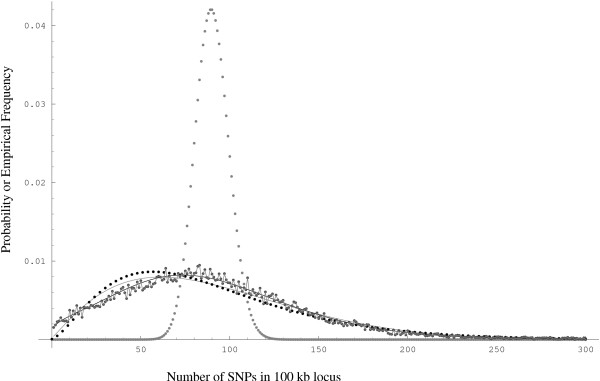
Fits of the homogeneous Poisson model (large gray dots), hierarchical Poisson model A (black dots) with *T *~ *G*(*γ*_1_, *γ*_2_) hierarchical Poisson model A' (gray line) with *T *= *λ *and *Λ *~ *B*(*β*_1_, *β*_2_), and hierarchical Poisson model B (black line) with *T *~ *G*(*β*_1_, *β*_2_) and *Λ *~ *B*(*β*_1_, *β*_2_) to the observed SNP density distribution (joined gray dots).

The likelihood function for each of the following hierarchical Poisson models was maximized with the Newton's method from several random initial conditions. We use the Akaike information criterion (*AIC*) [5] to make model comparisons. For a given model *AIC *= -2 log(*ML*) + 2*K*, where *ML *is the maximum likelihood value and *K *is the number of parameters in the model. In the hierarchical Poisson model A, we allow *T *~ *G*(*γ*_1_, *γ*_2_), while Λ is fixed at 1. The fit to the data (Figure 1) improved in comparison to the homogeneous Poisson fit which completely ignores the underlying ancestral recombination process. Thus, when the Gamma distribution is used to approximate the distribution of the sum of all branch lengths of the ancestral recombination graph (ARG-size) of a locus, the observed variance is better explained. Model A is mutationally homogeneous as Λ, the proxy for mutation rate, is fixed. In order to allow variation, a hierarchical Poisson model A' that restricts *T *to a constant parameter *λ *while allowing Λ to be Beta distributed (Λ ~ *B*(*β*_1_, *β*_2_)) was fit to the data. The fit was significantly better than that of model A. Thus, modeling heterogeneity in mutation rates, via the Beta distributed proxy Λ, across the different 100 kb loci gives better fits to the SNP density distribution. When we allowed both Λ to be Beta distributed and *T *to be Gamma distributed, we get the hierarchical Poisson model B. As shown in Figure 1, the fit is significantly better to the observed data when heterogeneities in both mutation and recombination are approximately accounted for through the proxies in model B. The results of the maximum likelihood (ML) analysis of these four Poisson models are summarized in Table 1. The first and second moments (μ^
 MathType@MTEF@5@5@+=feaafiart1ev1aaatCvAUfKttLearuWrP9MDH5MBPbIqV92AaeXatLxBI9gBaebbnrfifHhDYfgasaacH8akY=wiFfYdH8Gipec8Eeeu0xXdbba9frFj0=OqFfea0dXdd9vqai=hGuQ8kuc9pgc9s8qqaq=dirpe0xb9q8qiLsFr0=vr0=vr0dc8meaabaqaciaacaGaaeqabaqabeGadaaakeaaiiGacuWF8oqBgaqcaaaa@2E79@, and σ2_
 MathType@MTEF@5@5@+=feaafiart1ev1aaatCvAUfKttLearuWrP9MDH5MBPbIqV92AaeXatLxBI9gBaebbnrfifHhDYfgasaacH8akY=wiFfYdH8Gipec8Eeeu0xXdbba9frFj0=OqFfea0dXdd9vqai=hGuQ8kuc9pgc9s8qqaq=dirpe0xb9q8qiLsFr0=vr0=vr0dc8meaabaqaciaacaGaaeqabaqabeGadaaakeaadaqiaaqaaGGaciab=n8aZnaaCaaaleqabaGaeGOmaidaaaGccaGLcmaaaaa@3061@) under the maximum likelihood estimates are also shown for each model in the Table. Note that the means are almost the same but the variances vary considerably. If one wants a data-descriptive fit to the SNP density distribution, then Model B is a good candidate. With the arrival of more refined data (with counts in low-density regions as discussed later) one could consider further generalizations of such hierarchical Poisson models along the zero-inflated class [6], for instance, to obtain better descriptive fits. Unfortunately, the best-fitted parameters of such descriptive models lack any explicit biological interpretability, in terms of standard population-genetic models of reproduction. Guided by insights from these descriptive hierarchical Poisson models, we analyze the simplest population-genetic model of the neutral coalescent with an explicit accounting for heterogeneities in both mutation and recombination rates. We use a simulated maximum likelihood framework [7] for parameter estimation.

**Table 1:** Maximum likelihood analysis and comparison of Poisson models

Model	*T*	Λ	Maximum Likelihood Estimates	*ML*	AIC*
Poisson	*λ*	1	λ^ MathType@MTEF@5@5@+=feaafiart1ev1aaatCvAUfKttLearuWrP9MDH5MBPbIqV92AaeXatLxBI9gBaebbnrfifHhDYfgasaacH8akY=wiFfYdH8Gipec8Eeeu0xXdbba9frFj0=OqFfea0dXdd9vqai=hGuQ8kuc9pgc9s8qqaq=dirpe0xb9q8qiLsFr0=vr0=vr0dc8meaabaqaciaacaGaaeqabaqabeGadaaakeaaiiGacuWF7oaBgaqcaaaa@2E77@ = 90.2, μ^ MathType@MTEF@5@5@+=feaafiart1ev1aaatCvAUfKttLearuWrP9MDH5MBPbIqV92AaeXatLxBI9gBaebbnrfifHhDYfgasaacH8akY=wiFfYdH8Gipec8Eeeu0xXdbba9frFj0=OqFfea0dXdd9vqai=hGuQ8kuc9pgc9s8qqaq=dirpe0xb9q8qiLsFr0=vr0=vr0dc8meaabaqaciaacaGaaeqabaqabeGadaaakeaaiiGacuWF8oqBgaqcaaaa@2E79@ = 90.2, σ2_ MathType@MTEF@5@5@+=feaafiart1ev1aaatCvAUfKttLearuWrP9MDH5MBPbIqV92AaeXatLxBI9gBaebbnrfifHhDYfgasaacH8akY=wiFfYdH8Gipec8Eeeu0xXdbba9frFj0=OqFfea0dXdd9vqai=hGuQ8kuc9pgc9s8qqaq=dirpe0xb9q8qiLsFr0=vr0=vr0dc8meaabaqaciaacaGaaeqabaqabeGadaaakeaadaqiaaqaaGGaciab=n8aZnaaCaaaleqabaGaeGOmaidaaaGccaGLcmaaaaa@3061@ = 90.2	-616497	861964
A	*G*(*γ*_1_, *γ*_2_)	1	γ1_ MathType@MTEF@5@5@+=feaafiart1ev1aaatCvAUfKttLearuWrP9MDH5MBPbIqV92AaeXatLxBI9gBaebbnrfifHhDYfgasaacH8akY=wiFfYdH8Gipec8Eeeu0xXdbba9frFj0=OqFfea0dXdd9vqai=hGuQ8kuc9pgc9s8qqaq=dirpe0xb9q8qiLsFr0=vr0=vr0dc8meaabaqaciaacaGaaeqabaqabeGadaaakeaadaqiaaqaaGGaciab=n7aNnaaBaaaleaacqaIXaqmaeqaaaGccaGLcmaaaaa@3042@ = 2.7, γ2_ MathType@MTEF@5@5@+=feaafiart1ev1aaatCvAUfKttLearuWrP9MDH5MBPbIqV92AaeXatLxBI9gBaebbnrfifHhDYfgasaacH8akY=wiFfYdH8Gipec8Eeeu0xXdbba9frFj0=OqFfea0dXdd9vqai=hGuQ8kuc9pgc9s8qqaq=dirpe0xb9q8qiLsFr0=vr0=vr0dc8meaabaqaciaacaGaaeqabaqabeGadaaakeaadaqiaaqaaGGaciab=n7aNnaaBaaaleaacqaIYaGmaeqaaaGccaGLcmaaaaa@3044@ = 32.9, μ^ MathType@MTEF@5@5@+=feaafiart1ev1aaatCvAUfKttLearuWrP9MDH5MBPbIqV92AaeXatLxBI9gBaebbnrfifHhDYfgasaacH8akY=wiFfYdH8Gipec8Eeeu0xXdbba9frFj0=OqFfea0dXdd9vqai=hGuQ8kuc9pgc9s8qqaq=dirpe0xb9q8qiLsFr0=vr0=vr0dc8meaabaqaciaacaGaaeqabaqabeGadaaakeaaiiGacuWF8oqBgaqcaaaa@2E79@ = 90.2,	-186348	1670
			σ2_ MathType@MTEF@5@5@+=feaafiart1ev1aaatCvAUfKttLearuWrP9MDH5MBPbIqV92AaeXatLxBI9gBaebbnrfifHhDYfgasaacH8akY=wiFfYdH8Gipec8Eeeu0xXdbba9frFj0=OqFfea0dXdd9vqai=hGuQ8kuc9pgc9s8qqaq=dirpe0xb9q8qiLsFr0=vr0=vr0dc8meaabaqaciaacaGaaeqabaqabeGadaaakeaadaqiaaqaaGGaciab=n8aZnaaCaaaleqabaGaeGOmaidaaaGccaGLcmaaaaa@3061@ = 3049.7		
A'	*λ*	*B*(*β*_1_, *β*_2_)	λ^ MathType@MTEF@5@5@+=feaafiart1ev1aaatCvAUfKttLearuWrP9MDH5MBPbIqV92AaeXatLxBI9gBaebbnrfifHhDYfgasaacH8akY=wiFfYdH8Gipec8Eeeu0xXdbba9frFj0=OqFfea0dXdd9vqai=hGuQ8kuc9pgc9s8qqaq=dirpe0xb9q8qiLsFr0=vr0=vr0dc8meaabaqaciaacaGaaeqabaqabeGadaaakeaaiiGacuWF7oaBgaqcaaaa@2E77@ = 387.6, β1_ MathType@MTEF@5@5@+=feaafiart1ev1aaatCvAUfKttLearuWrP9MDH5MBPbIqV92AaeXatLxBI9gBaebbnrfifHhDYfgasaacH8akY=wiFfYdH8Gipec8Eeeu0xXdbba9frFj0=OqFfea0dXdd9vqai=hGuQ8kuc9pgc9s8qqaq=dirpe0xb9q8qiLsFr0=vr0=vr0dc8meaabaqaciaacaGaaeqabaqabeGadaaakeaadaqiaaqaaGGaciab=j7aInaaBaaaleaacqaIXaqmaeqaaaGccaGLcmaaaaa@303C@ = 2.17, β2_ MathType@MTEF@5@5@+=feaafiart1ev1aaatCvAUfKttLearuWrP9MDH5MBPbIqV92AaeXatLxBI9gBaebbnrfifHhDYfgasaacH8akY=wiFfYdH8Gipec8Eeeu0xXdbba9frFj0=OqFfea0dXdd9vqai=hGuQ8kuc9pgc9s8qqaq=dirpe0xb9q8qiLsFr0=vr0=vr0dc8meaabaqaciaacaGaaeqabaqabeGadaaakeaadaqiaaqaaGGaciab=j7aInaaBaaaleaacqaIYaGmaeqaaaGccaGLcmaaaaa@303E@ = 7.16,	-185869	714
			μ^ MathType@MTEF@5@5@+=feaafiart1ev1aaatCvAUfKttLearuWrP9MDH5MBPbIqV92AaeXatLxBI9gBaebbnrfifHhDYfgasaacH8akY=wiFfYdH8Gipec8Eeeu0xXdbba9frFj0=OqFfea0dXdd9vqai=hGuQ8kuc9pgc9s8qqaq=dirpe0xb9q8qiLsFr0=vr0=vr0dc8meaabaqaciaacaGaaeqabaqabeGadaaakeaaiiGacuWF8oqBgaqcaaaa@2E79@ = 90.1, σ2_ MathType@MTEF@5@5@+=feaafiart1ev1aaatCvAUfKttLearuWrP9MDH5MBPbIqV92AaeXatLxBI9gBaebbnrfifHhDYfgasaacH8akY=wiFfYdH8Gipec8Eeeu0xXdbba9frFj0=OqFfea0dXdd9vqai=hGuQ8kuc9pgc9s8qqaq=dirpe0xb9q8qiLsFr0=vr0=vr0dc8meaabaqaciaacaGaaeqabaqabeGadaaakeaadaqiaaqaaGGaciab=n8aZnaaCaaaleqabaGaeGOmaidaaaGccaGLcmaaaaa@3061@ = 2683.9		
B	*G*(*γ*_1_, *γ*_2_)	*B*(*β*_1_, *β*_2_)	γ1_ MathType@MTEF@5@5@+=feaafiart1ev1aaatCvAUfKttLearuWrP9MDH5MBPbIqV92AaeXatLxBI9gBaebbnrfifHhDYfgasaacH8akY=wiFfYdH8Gipec8Eeeu0xXdbba9frFj0=OqFfea0dXdd9vqai=hGuQ8kuc9pgc9s8qqaq=dirpe0xb9q8qiLsFr0=vr0=vr0dc8meaabaqaciaacaGaaeqabaqabeGadaaakeaadaqiaaqaaGGaciab=n7aNnaaBaaaleaacqaIXaqmaeqaaaGccaGLcmaaaaa@3042@ = 6.4, γ2_ MathType@MTEF@5@5@+=feaafiart1ev1aaatCvAUfKttLearuWrP9MDH5MBPbIqV92AaeXatLxBI9gBaebbnrfifHhDYfgasaacH8akY=wiFfYdH8Gipec8Eeeu0xXdbba9frFj0=OqFfea0dXdd9vqai=hGuQ8kuc9pgc9s8qqaq=dirpe0xb9q8qiLsFr0=vr0=vr0dc8meaabaqaciaacaGaaeqabaqabeGadaaakeaadaqiaaqaaGGaciab=n7aNnaaBaaaleaacqaIYaGmaeqaaaGccaGLcmaaaaa@3044@ = 19.0, β1_ MathType@MTEF@5@5@+=feaafiart1ev1aaatCvAUfKttLearuWrP9MDH5MBPbIqV92AaeXatLxBI9gBaebbnrfifHhDYfgasaacH8akY=wiFfYdH8Gipec8Eeeu0xXdbba9frFj0=OqFfea0dXdd9vqai=hGuQ8kuc9pgc9s8qqaq=dirpe0xb9q8qiLsFr0=vr0=vr0dc8meaabaqaciaacaGaaeqabaqabeGadaaakeaadaqiaaqaaGGaciab=j7aInaaBaaaleaacqaIXaqmaeqaaaGccaGLcmaaaaa@303C@ = 1.3,	-185511	0
			β2_ MathType@MTEF@5@5@+=feaafiart1ev1aaatCvAUfKttLearuWrP9MDH5MBPbIqV92AaeXatLxBI9gBaebbnrfifHhDYfgasaacH8akY=wiFfYdH8Gipec8Eeeu0xXdbba9frFj0=OqFfea0dXdd9vqai=hGuQ8kuc9pgc9s8qqaq=dirpe0xb9q8qiLsFr0=vr0=vr0dc8meaabaqaciaacaGaaeqabaqabeGadaaakeaadaqiaaqaaGGaciab=j7aInaaBaaaleaacqaIYaGmaeqaaaGccaGLcmaaaaa@303E@ = 0.46, μ^ MathType@MTEF@5@5@+=feaafiart1ev1aaatCvAUfKttLearuWrP9MDH5MBPbIqV92AaeXatLxBI9gBaebbnrfifHhDYfgasaacH8akY=wiFfYdH8Gipec8Eeeu0xXdbba9frFj0=OqFfea0dXdd9vqai=hGuQ8kuc9pgc9s8qqaq=dirpe0xb9q8qiLsFr0=vr0=vr0dc8meaabaqaciaacaGaaeqabaqabeGadaaakeaaiiGacuWF8oqBgaqcaaaa@2E79@ = 90.1, σ2_ MathType@MTEF@5@5@+=feaafiart1ev1aaatCvAUfKttLearuWrP9MDH5MBPbIqV92AaeXatLxBI9gBaebbnrfifHhDYfgasaacH8akY=wiFfYdH8Gipec8Eeeu0xXdbba9frFj0=OqFfea0dXdd9vqai=hGuQ8kuc9pgc9s8qqaq=dirpe0xb9q8qiLsFr0=vr0=vr0dc8meaabaqaciaacaGaaeqabaqabeGadaaakeaadaqiaaqaaGGaciab=n8aZnaaCaaaleqabaGaeGOmaidaaaGccaGLcmaaaaa@3061@ = 2538.2		

The last two columns give the maximum log likelihood values and the translated Akaike information criterion, AIC* = *AIC *- 371034.

### Population-Genetic Coalescent Mixture Models

A panmictic, Wright-Fisher, neutral coalescent model with a constant effective population size of 10,000 diploid individuals was assumed to simulate the distribution of the number of segregating sites at a locus of 100 kb evolving under an infinitely-many-sites mutation model using the C program ms [8]. The scaled product of the effective population size (*N*_*e*_) and the mutation rate per locus per generation (*μ*) is denoted by *θ *= 4*N*_*e*_*μ*. The recombination rate *r *is the probability of cross-over per generation between the ends of the locus being simulated and its scaled product with *N*_*e *_is denoted by *ρ *= 4*N*_*e*_*r*.

In the absence of recombination and with constant mutation rates, the distribution of SNPs is known to have an explicit form. The coalescent tree is identical for every nucleotide site in the locus in any given realization of the coalescent process of two samples. Since the rescaled time to the coalescent event and the mutation event are exponentially distributed with rates 1 and *θ*, respectively, the probability of a mutation event before the coalescent event is *θ*/(1 + *θ*). Thus, the probability of observing *x *mutations at our locus before the coalescent event is (*θ*/(1 + *θ*))^*x *^1/(1 + *θ*). In other words, the probability of observing *x *SNPs at a locus when *r *= 0 is geometrically distributed with parameter 1/(1 + *θ*).

It is also known that as the recombination rate at our locus approaches infinity, the distribution of SNPs approaches a Poisson distribution with parameter *θ*. This can be seen from the following argument. High levels of recombination assures that the coalescent tree at each site is independent of those at other sites. Thus, for a locus with *n *sites, the probability of observing *x *SNPs is (nx)(θn/(1+θn))x(1/(1+θn))n−x
 MathType@MTEF@5@5@+=feaafiart1ev1aaatCvAUfKttLearuWrP9MDH5MBPbIqV92AaeXatLxBI9gBaebbnrfifHhDYfgasaacH8akY=wiFfYdH8Gipec8Eeeu0xXdbba9frFj0=OqFfea0dXdd9vqai=hGuQ8kuc9pgc9s8qqaq=dirpe0xb9q8qiLsFr0=vr0=vr0dc8meaabaqaciaacaGaaeqabaqabeGadaaakeaadaqadaqaauaabeqaceaaaeaacqWGUbGBaeaacqWG4baEaaaacaGLOaGaayzkaaWaaeWaaeaadaWcgaqaamaalaaabaacciGae8hUdehabaGaemOBa4gaaaqaamaabmaabaGaeGymaeJaey4kaSYaaSaaaeaacqWF4oqCaeaacqWGUbGBaaaacaGLOaGaayzkaaaaaaGaayjkaiaawMcaamaaCaaaleqabaGaemiEaGhaaOWaaeWaaeaadaWcgaqaaiabigdaXaqaamaabmaabaGaeGymaeJaey4kaSYaaSaaaeaacqWF4oqCaeaacqWGUbGBaaaacaGLOaGaayzkaaaaaaGaayjkaiaawMcaamaaCaaaleqabaGaemOBa4MaeyOeI0IaemiEaGhaaaaa@4B2A@. For large loci, this binomial mass function is known to approximate *e*^-*θ*^*θ*^*x*^/*x*!, the Poisson mass function, as *n *→ ∞ and nθn/(1+θn)→θ
 MathType@MTEF@5@5@+=feaafiart1ev1aaatCvAUfKttLearuWrP9MDH5MBPbIqV92AaeXatLxBI9gBaebbnrfifHhDYfgasaacH8akY=wiFfYdH8Gipec8Eeeu0xXdbba9frFj0=OqFfea0dXdd9vqai=hGuQ8kuc9pgc9s8qqaq=dirpe0xb9q8qiLsFr0=vr0=vr0dc8meaabaqaciaacaGaaeqabaqabeGadaaakeaacqWGUbGBdaWcgaqaamaalaaabaacciGae8hUdehabaGaemOBa4gaaaqaamaabmaabaGaeGymaeJaey4kaSYaaSaaaeaacqWF4oqCaeaacqWGUbGBaaaacaGLOaGaayzkaaaaaiabgkziUkab=H7aXbaa@3B78@.

**Figure 2:**
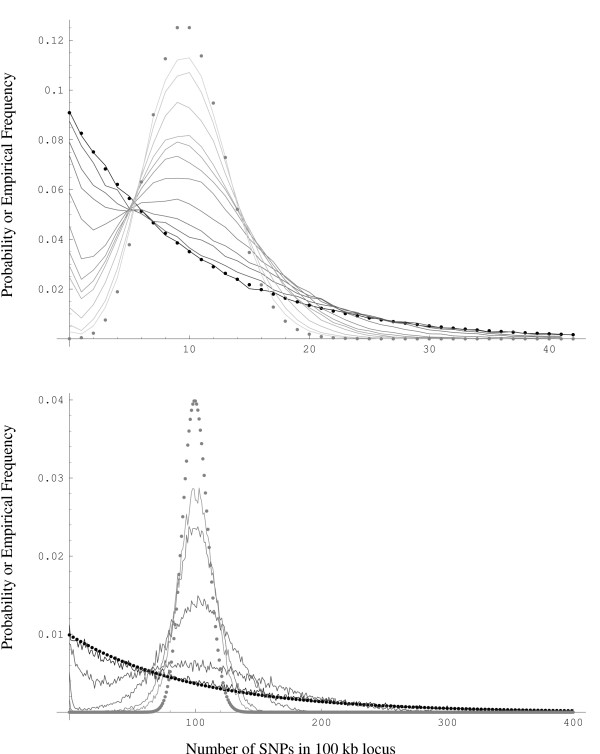
The distribution of SNP density in 100 kb morphs from the geometric distribution (black dots) towards the Poisson distribution (gray dots) as the scaled recombination rate *ρ *increases from 0 to 1000 in decreasing shades of gray for *θ *= 10 (top) and *θ *= 100 (bottom).

However, when the recombination rate is some intermediate value between the above two extremes no explicit forms are known for the SNP density. We use empirical estimates of the SNP density from a large number of simulations (typically 100,000). Figure 2 shows how the distribution of SNP density under our assumptions morphs from the geometric distribution (black dots) towards the Poisson distribution (grey dots) as the scaled recombination rate *ρ *increases from 0 to 1000 in decreasing shades of grey. This behavior is identical for any fixed value of *θ *except for a scale change.

**Figure 3:**
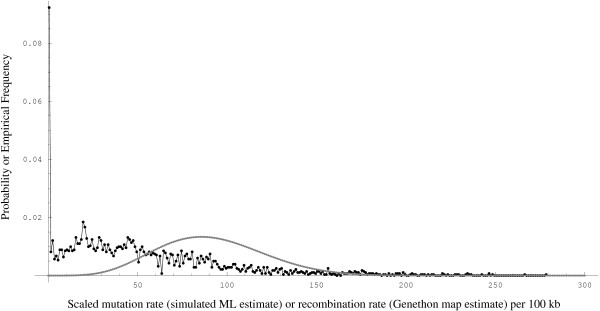
The distribution of the empirical estimates of the sex-averaged recombination rate in 100 kb segments of the human genome from the Genethon map (joined black dots) and wθi(6.7,14.9)
 MathType@MTEF@5@5@+=feaafiart1ev1aaatCvAUfKttLearuWrP9MDH5MBPbIqV92AaeXatLxBI9gBaebbnrfifHhDYfgasaacH8akY=wiFfYdH8Gipec8Eeeu0xXdbba9frFj0=OqFfea0dXdd9vqai=hGuQ8kuc9pgc9s8qqaq=dirpe0xb9q8qiLsFr0=vr0=vr0dc8meaabaqaciaacaGaaeqabaqabeGadaaakeaacqWG3bWDdaqhaaWcbaacciGae8hUde3aaSbaaWqaaiabdMgaPbqabaaaleaacqGGOaakcqaI2aGncqGGUaGlcqaI3aWncqGGSaalcqaIXaqmcqaI0aancqGGUaGlcqaI5aqocqGGPaqkaaaaaa@3AD6@, the maximum simulated likelihood estimate of the weights on *θ*_*i *_∈ Θ (gray line) for the coalescent mixture model.

The empirical estimates of the sex-averaged human recombination rates in 1 Mbp intervals based on Genethon [9], Marshfield [10] and deCODE [11] maps were downloaded from [12]. We intrapolated to obtain the estimates over 100 kb segments by assuming rate constancy over the 10 consecutive 100 kb segments that constitute the 1 Mbp segment for which an empirical estimate of the recombination rate were available. The empirical distribution of the sex-averaged human recombination rate in 100 kb intervals, based on Genethon map, as shown in Figure 3, is denoted by R_
 MathType@MTEF@5@5@+=feaafiart1ev1aaatCvAUfKttLearuWrP9MDH5MBPbIqV92AaeXatLxBI9gBaebbnrfifHhDYfgasaacH8akY=wiFfYdH8Gipec8Eeeu0xXdbba9frFj0=OqFfea0dXdd9vqai=hGuQ8kuc9pgc9s8qqaq=dirpe0xb9q8qiLsFr0=vr0=vr0dc8meaabaqaciaacaGaaeqabaqabeGadaaakeaadaqiaaqaaiabdkfasbGaayPadaaaaa@2E9B@. The strategy described in Methods was used to obtain a simulation-based empirical estimate of the SNP density distribution for each scaled mutation rate

*θ*_*i *_∈ Θ = {*θ*_1_, ⋯, *θ*_304_} = {0.001, 0.01, 0.1, 0.5, 1, 2, 3, 4, 5, ⋯, 298, 299, 300},

when the recombination rate was assumed to be distributed according to R_
 MathType@MTEF@5@5@+=feaafiart1ev1aaatCvAUfKttLearuWrP9MDH5MBPbIqV92AaeXatLxBI9gBaebbnrfifHhDYfgasaacH8akY=wiFfYdH8Gipec8Eeeu0xXdbba9frFj0=OqFfea0dXdd9vqai=hGuQ8kuc9pgc9s8qqaq=dirpe0xb9q8qiLsFr0=vr0=vr0dc8meaabaqaciaacaGaaeqabaqabeGadaaakeaadaqiaaqaaiabdkfasbGaayPadaaaaa@2E9B@. We denote this simulation-based estimate of the SNP density distribution for each *θ*_*i *_∈ Θ by S^R^,θi
 MathType@MTEF@5@5@+=feaafiart1ev1aaatCvAUfKttLearuWrP9MDH5MBPbIqV92AaeXatLxBI9gBaebbnrfifHhDYfgasaacH8akY=wiFfYdH8Gipec8Eeeu0xXdbba9frFj0=OqFfea0dXdd9vqai=hGuQ8kuc9pgc9s8qqaq=dirpe0xb9q8qiLsFr0=vr0=vr0dc8meaabaqaciaacaGaaeqabaqabeGadaaakeaacuWGtbWugaqcamaaBaaaleaacuWGsbGugaqcaiabcYcaSGGaciab=H7aXnaaBaaameaacqWGPbqAaeqaaaWcbeaaaaa@3384@. Note that S^R^,θi→SR^,θi
 MathType@MTEF@5@5@+=feaafiart1ev1aaatCvAUfKttLearuWrP9MDH5MBPbIqV92AaeXatLxBI9gBaebbnrfifHhDYfgasaacH8akY=wiFfYdH8Gipec8Eeeu0xXdbba9frFj0=OqFfea0dXdd9vqai=hGuQ8kuc9pgc9s8qqaq=dirpe0xb9q8qiLsFr0=vr0=vr0dc8meaabaqaciaacaGaaeqabaqabeGadaaakeaacuWGtbWugaqcamaaBaaaleaacuWGsbGugaqcaiabcYcaSGGaciab=H7aXnaaBaaameaacqWGPbqAaeqaaaWcbeaakiabgkziUkabdofatnaaBaaaleaacuWGsbGugaqcaiabcYcaSiab=H7aXnaaBaaameaacqWGPbqAaeqaaaWcbeaaaaa@3C37@, the true SNP density distribution, as the number of replicates (*N*) used to estimate it grows large. In practice, *N *was set at 100,000. A discretized and rescaled Beta density with parameters *α *and *β *was used to find the mixing weights for each *θ*_*i *_∈ Θ. Thus, for every ordered pair (*α*, *β*), the shape of the Beta density specified the mixing weights, as follows:

wθi(α,β)=∫i−1|Θ|i|Θ|Γ(α+β)Γ(α)Γ(β)λα−1(1−λ)β−1dλ
 MathType@MTEF@5@5@+=feaafiart1ev1aaatCvAUfKttLearuWrP9MDH5MBPbIqV92AaeXatLxBI9gBaebbnrfifHhDYfgasaacH8akY=wiFfYdH8Gipec8Eeeu0xXdbba9frFj0=OqFfea0dXdd9vqai=hGuQ8kuc9pgc9s8qqaq=dirpe0xb9q8qiLsFr0=vr0=vr0dc8meaabaqaciaacaGaaeqabaqabeGadaaakeaacqWG3bWDdaqhaaWcbaacciGae8hUde3aaSbaaWqaaiabdMgaPbqabaaaleaacqGGOaakcqWFXoqycqGGSaalcqWFYoGycqGGPaqkaaGccqGH9aqpdaWdXaqaamaalaaabaGaeu4KdCKaeiikaGIae8xSdeMaey4kaSIae8NSdiMaeiykaKcabaGaeu4KdCKaeiikaGIae8xSdeMaeiykaKIaeu4KdCKaeiikaGIae8NSdiMaeiykaKcaaaWcbaWaaSaaaeaacqWGPbqAcqGHsislcqaIXaqmaeaadaabdaqaaiabfI5arbGaay5bSlaawIa7aaaaaeaadaWcaaqaaiabdMgaPbqaamaaemaabaGaeuiMdefacaGLhWUaayjcSdaaaaqdcqGHRiI8aOGae83UdW2aaWbaaSqabeaacqWFXoqycqGHsislcqaIXaqmaaGccqGGOaakcqaIXaqmcqGHsislcqWF7oaBcqGGPaqkdaahaaWcbeqaaiab=j7aIjabgkHiTiabigdaXaaakiabdsgaKjab=T7aSbaa@6A87@

where, |Θ| = 304 is the cardinality or size of the set Θ. Such (*α*, *β*)-specified wθi(α,β)
 MathType@MTEF@5@5@+=feaafiart1ev1aaatCvAUfKttLearuWrP9MDH5MBPbIqV92AaeXatLxBI9gBaebbnrfifHhDYfgasaacH8akY=wiFfYdH8Gipec8Eeeu0xXdbba9frFj0=OqFfea0dXdd9vqai=hGuQ8kuc9pgc9s8qqaq=dirpe0xb9q8qiLsFr0=vr0=vr0dc8meaabaqaciaacaGaaeqabaqabeGadaaakeaacqWG3bWDdaqhaaWcbaacciGae8hUde3aaSbaaWqaaiabdMgaPbqabaaaleaacqGGOaakcqWFXoqycqGGSaalcqWFYoGycqGGPaqkaaaaaa@3768@'s were used to weigh the corresponding S^R^,θi
 MathType@MTEF@5@5@+=feaafiart1ev1aaatCvAUfKttLearuWrP9MDH5MBPbIqV92AaeXatLxBI9gBaebbnrfifHhDYfgasaacH8akY=wiFfYdH8Gipec8Eeeu0xXdbba9frFj0=OqFfea0dXdd9vqai=hGuQ8kuc9pgc9s8qqaq=dirpe0xb9q8qiLsFr0=vr0=vr0dc8meaabaqaciaacaGaaeqabaqabeGadaaakeaacuWGtbWugaqcamaaBaaaleaacuWGsbGugaqcaiabcYcaSGGaciab=H7aXnaaBaaameaacqWGPbqAaeqaaaWcbeaaaaa@3384@'s in order to obtain a finite mixture of the form ∑θi∈Θwθi(α,β)S^R^,θi
 MathType@MTEF@5@5@+=feaafiart1ev1aaatCvAUfKttLearuWrP9MDH5MBPbIqV92AaeXatLxBI9gBaebbnrfifHhDYfgasaacH8akY=wiFfYdH8Gipec8Eeeu0xXdbba9frFj0=OqFfea0dXdd9vqai=hGuQ8kuc9pgc9s8qqaq=dirpe0xb9q8qiLsFr0=vr0=vr0dc8meaabaqaciaacaGaaeqabaqabeGadaaakeaadaaeqaqaaiabdEha3naaDaaaleaaiiGacqWF4oqCdaWgaaadbaGaemyAaKgabeaaaSqaaiabcIcaOiab=f7aHjabcYcaSiab=j7aIjabcMcaPaaaaeaacqWF4oqCdaWgaaadbaGaemyAaKgabeaaliabgIGiolabfI5arbqab0GaeyyeIuoakiqbdofatzaajaWaaSbaaSqaaiqbdkfaszaajaGaeiilaWIae8hUde3aaSbaaWqaaiabdMgaPbqabaaaleqaaaaa@4655@. A simulated likelihood function of *α *and *β *was thus constructed for the given SNP data *X *= (*x*_0_, ∈, *x*_*n*_), as follows,

∏j=0n∑θi∈Θwθi(α,β)S^R^,θi(xj)
 MathType@MTEF@5@5@+=feaafiart1ev1aaatCvAUfKttLearuWrP9MDH5MBPbIqV92AaeXatLxBI9gBaebbnrfifHhDYfgasaacH8akY=wiFfYdH8Gipec8Eeeu0xXdbba9frFj0=OqFfea0dXdd9vqai=hGuQ8kuc9pgc9s8qqaq=dirpe0xb9q8qiLsFr0=vr0=vr0dc8meaabaqaciaacaGaaeqabaqabeGadaaakeaadaqeWbqaamaaqafabaGaem4DaC3aa0baaSqaaGGaciab=H7aXnaaBaaameaacqWGPbqAaeqaaaWcbaGaeiikaGIae8xSdeMaeiilaWIae8NSdiMaeiykaKcaaOGafm4uamLbaKaadaWgaaWcbaGafmOuaiLbaKaacqGGSaalcqWF4oqCdaWgaaadbaGaemyAaKgabeaaaSqabaGccqGGOaakcqWG4baEdaWgaaWcbaGaemOAaOgabeaakiabcMcaPaWcbaGae8hUde3aaSbaaWqaaiabdMgaPbqabaWccqGHiiIZcqqHyoquaeqaniabggHiLdaaleaacqWGQbGAcqGH9aqpcqaIWaamaeaacqWGUbGBa0Gaey4dIunaaaa@524F@

**Figure 4:**
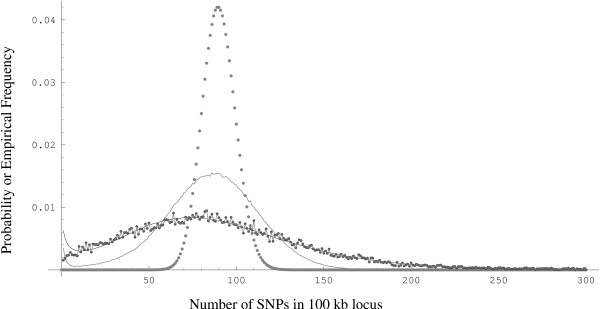
The SNP density distribution (joined gray dots), Poisson distribution with mean 90 (large gray dots), simulated distribution of SNPs with *ρ *= *θ *= 90 (gray line), and the Maximum Simulated Likelihood estimate from the coalescent simulations with *ρ *~ R_
 MathType@MTEF@5@5@+=feaafiart1ev1aaatCvAUfKttLearuWrP9MDH5MBPbIqV92AaeXatLxBI9gBaebbnrfifHhDYfgasaacH8akY=wiFfYdH8Gipec8Eeeu0xXdbba9frFj0=OqFfea0dXdd9vqai=hGuQ8kuc9pgc9s8qqaq=dirpe0xb9q8qiLsFr0=vr0=vr0dc8meaabaqaciaacaGaaeqabaqabeGadaaakeaadaqiaaqaaiabdkfasbGaayPadaaaaa@2E9B@*θ*_*i *_~ wθi(6.7,14.9)
 MathType@MTEF@5@5@+=feaafiart1ev1aaatCvAUfKttLearuWrP9MDH5MBPbIqV92AaeXatLxBI9gBaebbnrfifHhDYfgasaacH8akY=wiFfYdH8Gipec8Eeeu0xXdbba9frFj0=OqFfea0dXdd9vqai=hGuQ8kuc9pgc9s8qqaq=dirpe0xb9q8qiLsFr0=vr0=vr0dc8meaabaqaciaacaGaaeqabaqabeGadaaakeaacqWG3bWDdaqhaaWcbaacciGae8hUde3aaSbaaWqaaiabdMgaPbqabaaaleaacqGGOaakcqaI2aGncqGGUaGlcqaI3aWncqGGSaalcqaIXaqmcqaI0aancqGGUaGlcqaI5aqocqGGPaqkaaaaaa@3AD6@ and (black line).

We used the Newton's method to find the maximum simulated likelihood (*MSL*) estimates α^
 MathType@MTEF@5@5@+=feaafiart1ev1aaatCvAUfKttLearuWrP9MDH5MBPbIqV92AaeXatLxBI9gBaebbnrfifHhDYfgasaacH8akY=wiFfYdH8Gipec8Eeeu0xXdbba9frFj0=OqFfea0dXdd9vqai=hGuQ8kuc9pgc9s8qqaq=dirpe0xb9q8qiLsFr0=vr0=vr0dc8meaabaqaciaacaGaaeqabaqabeGadaaakeaaiiGacuWFXoqygaqcaaaa@2E62@ = 6.7 and β^
 MathType@MTEF@5@5@+=feaafiart1ev1aaatCvAUfKttLearuWrP9MDH5MBPbIqV92AaeXatLxBI9gBaebbnrfifHhDYfgasaacH8akY=wiFfYdH8Gipec8Eeeu0xXdbba9frFj0=OqFfea0dXdd9vqai=hGuQ8kuc9pgc9s8qqaq=dirpe0xb9q8qiLsFr0=vr0=vr0dc8meaabaqaciaacaGaaeqabaqabeGadaaakeaaiiGacuWFYoGygaqcaaaa@2E64@ = 14.9 (*MSL *= -185555). We also did a least-squares fit of the observed to the predicted densities and found comparable estimates. Empirical estimates of the sex-averaged recombination rates from deCODE, and Marshfield maps were also used in a similar analysis. Comparable estimates were obtained under a reasonably good fit (*MSL *= -185558) with the deCODE map whose empirical CDF resembles that of the Genethon Map. However, an analysis with the Marshfield map yielded a poorer fit (*MSL *= -186007). Figure 4 summarizes the fits to the observed SNP data while Figure 3 shows the marginal density of *ρ *from the Genethon map and the marginal density of *θ *under the maximum simulated likelihood estimates (α^
 MathType@MTEF@5@5@+=feaafiart1ev1aaatCvAUfKttLearuWrP9MDH5MBPbIqV92AaeXatLxBI9gBaebbnrfifHhDYfgasaacH8akY=wiFfYdH8Gipec8Eeeu0xXdbba9frFj0=OqFfea0dXdd9vqai=hGuQ8kuc9pgc9s8qqaq=dirpe0xb9q8qiLsFr0=vr0=vr0dc8meaabaqaciaacaGaaeqabaqabeGadaaakeaaiiGacuWFXoqygaqcaaaa@2E62@ = 6.7, β^
 MathType@MTEF@5@5@+=feaafiart1ev1aaatCvAUfKttLearuWrP9MDH5MBPbIqV92AaeXatLxBI9gBaebbnrfifHhDYfgasaacH8akY=wiFfYdH8Gipec8Eeeu0xXdbba9frFj0=OqFfea0dXdd9vqai=hGuQ8kuc9pgc9s8qqaq=dirpe0xb9q8qiLsFr0=vr0=vr0dc8meaabaqaciaacaGaaeqabaqabeGadaaakeaaiiGacuWFYoGygaqcaaaa@2E64@ = 14.9) with mean, variance, and standard deviation given by 90.7, 876.1, and 29.6, respectively. Among the three coarse-scaled maps of the *empirical *estimates of the sex-averaged human recombination rates, the Genethon map gave the best fit to our observed SNP density distribution data.

## Discussion

Another study [13] claimed to have achieved a good fit to single reads with 0, 1, 2, 3, or 4 SNPs, by accounting for mutational heterogeneity and genealogical variability in a different manner. They partitioned the genome into 200 kb bins, and selected a single read from each bin. They calculated the observed GC content of the bin, and from a regression of GC content on nucleotide diversity across the whole genome, they calculated an expected diversity given the local GC content of each bin induced by the exponentially distributed coalescent time for samples of size 2 in the absence of recombination. However, when the full bin size of 100 kb were used [2], the SNP count ranged to more than 100 per bin. Because many neighboring reads have shared genealogies, the magnitude of variability from bin to bin is much greater, and the power to detect this heterogeneity is far greater. Thus, the latter study [2] found that the coalescent in the presence of recombination fit the observed SNP density better than the coalescent without recombination. The model employed in the former study [13] fits without recombination only because the power is so low to detect a departure and because there are correspondingly fewer recombination events expected within single reads vs. 100 kb bins. Using the data of SNP counts in 100 kb bins in this study, we find that the coalescent with heterogeneities in recombination as well as mutation gives substantially better fits than the coalescent with a constant rate of recombination and mutation. We have shown that by invoking heterogeneities in mutation and recombination rates, one can better explain the observed variation in SNP density across two randomly sampled 100 kb segments of human chromosomes. Descriptive fits by means of hierarchical Poisson models, as well as population-genetic fits by means of coalescent mixture models, significantly improved when heterogeneities in recombination as well as mutation rates were accounted for. The coalescent mixture model does not completely fit the data in the most interesting region, namely, the segments with the least SNP density. This is partly due to the filtering strategy used to obtain the data. Since there were considerable gaps in the alignments for several bins, there was an overestimation of bins with 0 SNPs. Thus, these bins were ignored from the analysis. Were low SNP counts from such currently ignored bins made available from a high-resolution alignment, a similar analysis would reveal the poorer fits of the descriptive hierarchical Poisson models employed here, unless they are further generalized to allow for a larger mass at 0 through the zero-inflated class [6], for instance. If one's objective is to produce a descriptive fit to our observed SNP density distribution, then the hierarchical model B is clearly preferable to all the models considered in this study due to its strikingly high likelihood value. However, if one wanted a population-genetic model with biologically interpretable parameters to fit the same data, then the best fitted coalescent mixture model with the Genethon recombination map is preferable.

It is important to bear in mind that the distribution of *T *will be affected not only by recombination rate but also by population structure and demography. Likewise, the distribution of Λ and *T *will be affected by the complex interaction between various DNA damage and repair pathways that ultimately lead to various types of mutational and recombinational events [[1], for e.g.]. Moreover, the action of selection will simultaneously affect both the distribution of *T *and Λ about the selected site(s). However, since only a small percentage of the genome is expected to be affected by recent selective sweeps, the overall SNP density distribution should not be significantly affected by such selective events. Thus, our MSL estimate of the genomic variation in *θ*, based on the Genethon map, is under the standard neutral coalescent that allows for recombinational and mutational rate heterogeneity across the genome. The true genomic variation in *θ *can also be affected by several other confounded historical factors including selection, population structure, and demography, besides genomic variation in mutation rate. All these confounded historical factors can be seen as alternative hypotheses to the null hypothesis of our coalescent mixture model for the SNP density distribution, i.e., the standard neutral coalescent with genomic heterogeneity in recombination and mutation rates.

## Conclusion

As high resolution data for larger samples become available at a genomic scale, one can use such simulated ML methods (with appropriate sample sizes) to get the null distributions of various test statistics while accounting for heterogeneities in recombination rates (from empirical maps or finer-scaled inferred maps) and mutation rates (from the informative phylogenomic constraints imposed by additional ape genomes). Such empirically observable phenomena should be incorporated into the null hypothesis when more complex models with unobserved historical phenomena, such as population dynamics, population structure, and/or natural selection are tested in humans at the genomic scale. Current scans of the human genome tend to underestimate the costs of ignoring the empirically observable heterogeneities under the null hypothesis.

## Methods

In the hierarchical Poisson scheme, heterogeneities are modeled by the following Gamma and Beta probability density functions (*PDF*s),

T~G(γ1,γ2),where,PDF(t)=1Γ(γ1)γ2γ1tγ1−1exp⁡(−tγ2),0≤t<∞,γ1,γ2>0,Λ~B(β1,β2),where,PDF(λ)=Γ(β1+β2)Γ(β1)Γ(β2)λβ1−1(1−λ)β2−1,0≤λ≤1,β1,β2>0.
 MathType@MTEF@5@5@+=feaafiart1ev1aaatCvAUfKttLearuWrP9MDH5MBPbIqV92AaeXatLxBI9gBaebbnrfifHhDYfgasaacH8akY=wiFfYdH8Gipec8Eeeu0xXdbba9frFj0=OqFfea0dXdd9vqai=hGuQ8kuc9pgc9s8qqaq=dirpe0xb9q8qiLsFr0=vr0=vr0dc8meaabaqaciaacaGaaeqabaqabeGadaaakeaafaqabeabcaaaaeaacqWGubavcqGG+bGFcqWGhbWrcqGGOaakiiGacqWFZoWzdaWgaaWcbaGaeGymaedabeaakiabcYcaSiab=n7aNnaaBaaaleaacqaIYaGmaeqaaOGaeiykaKIaeiilaWcabaaabaGamaiGaaae=h4DaCNamaiGaaae=hiAaGMamaiGaaae=hyzauMamaiGaaae=hOCaiNamaiGaaae=hyzauMamaiGaaae=lilaWIamaiGaaae=piuaaLamaiGaaae=piraqKamaiGaaae=pOrayKamaiGaaae=likaGIamaiGaaae=piDaqNamaiGaaae=lykaKcabaqbaeqabiqaaaqaaiabg2da9maalaaabaGaeGymaedabaGaeu4KdCKaeiikaGIae83SdC2aaSbaaSqaaiabigdaXaqabaGccqGGPaqkcqWFZoWzdaqhaaWcbaGaeGOmaidabaGae83SdC2aaSbaaWqaaiabigdaXaqabaaaaaaakiabdsha0naaCaaaleqabaGae83SdC2aaSbaaWqaaiabigdaXaqabaWccqGHsislcqaIXaqmaaGccyGGLbqzcqGG4baEcqGGWbaCdaqadaqaaiabgkHiTmaalaaabaGaemiDaqhabaGae83SdC2aaSbaaSqaaiabikdaYaqabaaaaaGccaGLOaGaayzkaaGaeiilaWcabaGaeGimaaJaeyizImQaemiDaqNaeyipaWJaeyOhIuQaeiilaWIae83SdC2aaSbaaSqaaiabigdaXaqabaGccqGGSaalcqWFZoWzdaWgaaWcbaGaeGOmaidabeaakiabg6da+iabicdaWiabcYcaSaaaaeaacqqHBoatcqGG+bGFcqWGcbGqcqGGOaakcqWFYoGydaWgaaWcbaGaeGymaedabeaakiabcYcaSiab=j7aInaaBaaaleaacqaIYaGmaeqaaOGaeiykaKIaeiilaWcabaaabaGamaiGaaae=h4DaCNamaiGaaae=hiAaGMamaiGaaae=hyzauMamaiGaaae=hOCaiNamaiGaaae=hyzauMamaiGaaae=lilaWIamaiGaaae=piuaaLamaiGaaae=piraqKamaiGaaae=pOrayKamaiGaaae=likaGIamaiGaaae==3UdWMamaiGaaae=lykaKcabaqbaeqabiqaaaqaaiabg2da9maalaaabaGaeu4KdCKaeiikaGIae8NSdi2aaSbaaSqaaiabigdaXaqabaGccqGHRaWkcqWFYoGydaWgaaWcbaGaeGOmaidabeaakiabcMcaPaqaaiabfo5ahjabcIcaOiab=j7aInaaBaaaleaacqaIXaqmaeqaaOGaeiykaKIaeu4KdCKaeiikaGIae8NSdi2aaSbaaSqaaiabikdaYaqabaGccqGGPaqkaaGae83UdW2aaWbaaSqabeaacqWFYoGydaWgaaadbaGaeGymaedabeaaliabgkHiTiabigdaXaaakiabcIcaOiabigdaXiabgkHiTiab=T7aSjabcMcaPmaaCaaaleqabaGae8NSdi2aaSbaaWqaaiabikdaYaqabaWccqGHsislcqaIXaqmaaGccqGGSaalaeaacqaIWaamcqGHKjYOcqWF7oaBcqGHKjYOcqaIXaqmcqGGSaalcqWFYoGydaWgaaWcbaGaeGymaedabeaakiabcYcaSiab=j7aInaaBaaaleaacqaIYaGmaeqaaOGaeyOpa4JaeGimaaJaeiOla4caaaaaaaa@FEA1@

The following strategy was used to obtain a simulation-based empirical estimate of the SNP density distribution for each scaled mutation rate

*θ*_*i *_∈ Θ = {*θ*_1_, ⋯, *θ*_304_} = {0.001, 0.01, 0.1, 0.5, 1, 2, 3, 4, 5, ⋯, 298, 299, 300},

when the recombination rate was assumed to be distributed according to

R_
 MathType@MTEF@5@5@+=feaafiart1ev1aaatCvAUfKttLearuWrP9MDH5MBPbIqV92AaeXatLxBI9gBaebbnrfifHhDYfgasaacH8akY=wiFfYdH8Gipec8Eeeu0xXdbba9frFj0=OqFfea0dXdd9vqai=hGuQ8kuc9pgc9s8qqaq=dirpe0xb9q8qiLsFr0=vr0=vr0dc8meaabaqaciaacaGaaeqabaqabeGadaaakeaadaqiaaqaaiabdkfasbGaayPadaaaaa@2E9B@.

1. for each *θ*_*i *_∈ Θ, repeat N times:

(a) sample a *ρ *according to

R_
 MathType@MTEF@5@5@+=feaafiart1ev1aaatCvAUfKttLearuWrP9MDH5MBPbIqV92AaeXatLxBI9gBaebbnrfifHhDYfgasaacH8akY=wiFfYdH8Gipec8Eeeu0xXdbba9frFj0=OqFfea0dXdd9vqai=hGuQ8kuc9pgc9s8qqaq=dirpe0xb9q8qiLsFr0=vr0=vr0dc8meaabaqaciaacaGaaeqabaqabeGadaaakeaadaqiaaqaaiabdkfasbGaayPadaaaaa@2E9B@

(b) simulate the coalescent according to *ρ *and *θ*_*i *_[4,8]

(c) record the number of SNPs

2. Obtain the empirical distribution of SNP density for the given *θ*_*i *_when *ρ *~

R_
 MathType@MTEF@5@5@+=feaafiart1ev1aaatCvAUfKttLearuWrP9MDH5MBPbIqV92AaeXatLxBI9gBaebbnrfifHhDYfgasaacH8akY=wiFfYdH8Gipec8Eeeu0xXdbba9frFj0=OqFfea0dXdd9vqai=hGuQ8kuc9pgc9s8qqaq=dirpe0xb9q8qiLsFr0=vr0=vr0dc8meaabaqaciaacaGaaeqabaqabeGadaaakeaadaqiaaqaaiabdkfasbGaayPadaaaaa@2E9B@

## Authors' contributions

AGC posed the question, RS and RTD made simple models, RS implemented the models and all three authors edited the manuscript.
